# Pandemic-Driven Development of a Medical-Grade, Economic and Decentralized Applicable Polyolefin Filament for Additive Fused Filament Fabrication

**DOI:** 10.3390/molecules25245929

**Published:** 2020-12-15

**Authors:** Felix Burkhardt, Carl G. Schirmeister, Christian Wesemann, Massimo Nutini, Stefano Pieralli, Erik H. Licht, Marc Metzger, Frederik Wenz, Rolf Mülhaupt, Benedikt C. Spies

**Affiliations:** 1 Center for Dental Medicine, Department of Prosthetic Dentistry, Faculty of Medicine, University of Freiburg, Hugstetter Str. 55, 79106 Freiburg, Germany; felix.burkhardt@uniklinik-freiburg.de (F.B.); christian.wesemann@uniklinik-freiburg.de (C.W.); stefano.pieralli@uniklinik-freiburg.de (S.P.); 2 Freiburg Materials Research Center FMF and Institute for Macromolecular Chemistry, Albert-Ludwigs-University Freiburg, Stefan-Meier-Str. 21, 79104 Freiburg, Germany; carl.schirmeister@fmf.uni-freiburg.de (C.G.S.); rolf.muelhaupt@makro.uni-freiburg.de (R.M.); 3 Basell Sales & Marketing B.V., LyondellBasell Industries, Industriepark Höchst, 65926 Frankfurt am Main, Germany; Erik.Licht@lyondellbasell.com; 4 Basell Poliolefine Italia Srl, LyondellBasell Industries, P. le Privato G. Donegani 12, 44122 Ferrara, Italy; massimo.nutini@lyb.com; 5 Center of Dental Medicine, Department of Oral and Maxillofacial Surgery, University of Freiburg, Hugstetter Str. 55, 79106 Freiburg, Germany; marc.metzger@uniklinik-freiburg.de; 6 Board of Directors, Faculty of Medicine, University of Freiburg, Hugstetter Str. 55, 79106 Freiburg, Germany; frederik.wenz@uniklinik-freiburg.de; 7 Sustainability Center Freiburg, Ecker-Str. 4, 79104 Freiburg, Germany

**Keywords:** COVID-19, additive manufacturing, fused filament fabrication, fused deposition modeling, 3D printing, polypropylene, copolymer, medical grade, personal protective equipment

## Abstract

A polyolefin with certified biocompatibility according to USP class VI was used by our group as feedstock for filament-based 3D printing to meet the highest medical standards in order to print personal protective equipment for our university hospital during the ongoing pandemic. Besides the chemical resistance and durability, as well as the ability to withstand steam sterilization, this polypropylene (PP) copolymer is characterized by its high purity, as achieved by highly efficient and selective catalytic polymerization. As the PP copolymer is suited to be printed with all common printers in fused filament fabrication (FFF), it offers an eco-friendly cost–benefit ratio, even for large-scale production. In addition, a digital workflow was established focusing on common desktop FFF printers in the medical sector. It comprises the simulation-based optimization of personalized print objects, considering the inherent material properties such as warping tendency, through to validation of the process chain by 3D scanning, sterilization, and biocompatibility analysis of the printed part. This combination of digital data processing and 3D printing with a sustainable and medically certified material showed great promise in establishing decentralized additive manufacturing in everyday hospital life to meet peaks in demand, supply bottlenecks, and enhanced personalized patient treatment.

## 1. Introduction

The severe acute respiratory syndrome coronavirus 2 (SARS-CoV-2) rapidly spread from China in December 2019, causing the ongoing coronavirus pandemic (COVID-19), which led to an international public health emergency [1]. The virus is mainly transmitted respiratory via fluid particles containing the virus, which are formed when breathing, speaking, and coughing [2]. In the medical sector, aerosol-generating otolaryngology procedures such as intubation or bronchoscopy pose an increased risk of infection [3]. Moreover, dental treatment along with water cooling-induced aerosol production is known to create a highly virulent environment for treating professionals [4]. As long as there are no effective pharmacological interventions or vaccinations available, infection prevention with personal protective equipment (PPE) seems inevitable [5]. In this context, it was shown that PPE consisting of a mouth/nose mask, face shield, and goggles reduced the risk of inhalation for healthcare workers treating COVID-19 patients [6,7]. Due to the high number of PPE needed worldwide during the emerging pandemic and difficulties in global supply chains, a shortage of PPE for health care workers resulted, as widely known, which may lead to inadequate protection. Therefore, in times of crisis, a decentralized, globally available, flexible, and reliable in-house production method for PPE and other medical devices with a cost-efficient and sustainable material is of great interest. Thus, the manufacturing process should require minimal technical knowledge and involve as few steps as possible. For this purpose, additive manufacturing (AM) using three-dimensional (3D) printers is particularly suitable. The focus lies on the use of a material that is easy to print, can be used in a health-critical environment, and also has the appropriate certifications and approvals.

## 2. Additive Manufacturing (AM)

In additive manufacturing (AM), computer-designed or scanned virtual 3D objects are transformed via digital slicing into real 3D objects by adding layers on top of each other during the 3D printing process. In contrast to subtractive or formative manufacturing, the objects do not require time-consuming production of molds or machining and are built in a single process step. High-tech process chains and vulnerable global transport routes are being replaced by modern decentralized production facilities that manufacture parts adapted to local conditions and even personalized for the patient or customer. Instead of finished parts, there is only a global but still instantaneous data exchange, which provides the template for required spare parts or, for example, PPE.

In the medical sector, stereolithography (SLA) and digital light processing (DLP) are the most commonly used AM techniques. Both are based on liquid photopolymer resins, which are cured with ultraviolet laser radiation [8]. This enables the manufacturing of highly accurate objects, e.g., surgical guides or hearing aids [9]. However, extensive post-processing such as cleaning and washing procedures as well as additional light-curing is necessary. This requires further equipment and materials, thereby increasing the costs. Besides, transport, storage, and aging of the photosensitive resins are challenging and require the handling of trained personnel, which may lead to bottlenecks during a pandemic or in remote areas. Even in everyday use in the healthcare sector, these boundary conditions and post-processing steps require additional personnel expenditure, more complex certification, and, due to the lack of automation and additional process steps, also have a higher risk of material and component defects. Powder-based 3D printing technologies, such as selective laser sintering (SLS) or multi-jet fusion (MJF), also require post-processing of the printed parts, profound know-how, and a sophisticated cost-intensive technology to safely handle the powders.

**Figure 1:**
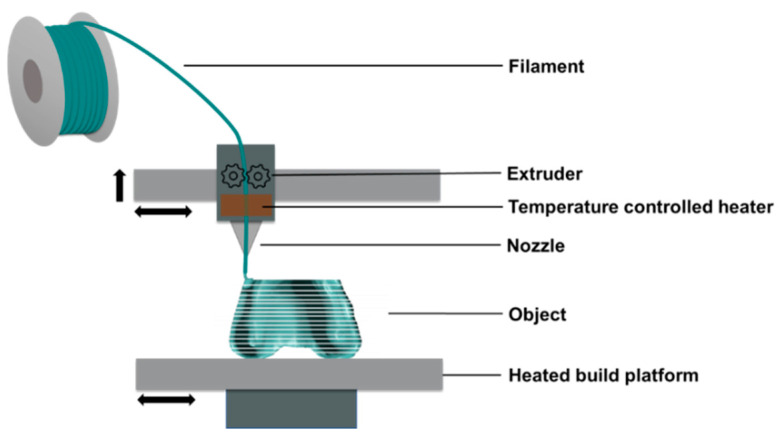
Fused filament fabrication (FFF), often referred to as fused deposition modeling (FDM), invented by Scott Crump at Stratasys Inc. (Eden Prairie, MN, USA), comprises a tempered extrusion printing head for deposition of molten thermoplastic material stored in feedstocks containing filaments.

As an alternative, fused filament fabrication (FFF), also known under the trademark term fused deposition modeling (FDM), can be used to produce 3D objects cost-effectively and with little technical effort (Figure 1) [8]. Since FFF technology was pioneered by Scott Crump at Stratasys Inc. in 1989 [10], it has been used not only in research, but also in manufacturing, rapid prototyping, medicine, and the hobby sector, leading to a growth at annual double-digit rates. The robust and cost-competitive design of FFF is the main reason for its great success all over the world. In FFF, a thermoplastic material is fed into a heated nozzle, where it is melted and then deposited layer by layer to create finished parts that do not require cleaning or curing steps.

The reliable FFF process is highly suitable for everyday use in the medical sector. From implant printing under clean room conditions to the just-in-time printing of personalized aids on desktop printers in clinics and medical practices, countless applications of FFF are possible to improve patient care. This includes the drug loading with filaments [11,12,13], which offers new possibilities for the production of implants with an intrinsic drug depot to tailor patient care comprehensively [14]. In addition, FFF printing can be used to produce skin patches containing an appropriate therapeutic drug concentration with the desired release profile [15]. Among other printing techniques, FFF can be applied to produce medical instruments for diagnostics and surgery [15]. Although this technology still faces many challenges, such as contamination with debris or the ability to sterilize the printed objects, it offers many new opportunities in terms of cost-effective customized instruments. In the dental sector, for example, FFF printing can be used to produce surgical guides for implantation. Although the precision of FFF-printed objects is currently under discussion, it was observed that FFF-printed surgical guides made of polylactic acid (PLA) lead to an accuracy comparable to SLA-printed guides [16]. Especially in periods of crisis, when a high demand for protection and medical equipment means they need to be produced in-house within a short period of time, a cost-effective, straight-forward procedure is considered advantageous. Therefore, FFF printing was used to produce PPE during the ongoing pandemic to protect health care workers [17,18].

## 3. Materials for AM

Besides the choice of the appropriate printing process, the material selection is of decisive importance. The basic material requirements for the application in FFF are a low warpage tendency, adhesion to the build plate without impairing facile detachment, and a fast and stable fusion of the deposited strands and layers. Only if these requirements are met simultaneously will comparable mechanical properties and dimensional accuracy to established manufacturing techniques be achieved.

To avoid warpage, the most common polymers applied in FFF are amorphous, such as acrylonitrile-butadiene-styrene copolymers (ABS), transparent polycarbonates (PC), polyethylene terephthalate glycol-modified (PETG), or semi-crystalline thermoplastics with rather low crystallization rates and a low crystallinity, such as polylactic acid [8,19,20]. Other products include soft and flexible thermoplastic polyurethanes (TPU) and water-soluble polyvinyl alcohols. The wide range of materials now available for extrusion-based 3D printing thus goes far beyond the choice offered by other 3D printing techniques. It also includes mechanically improved blends and composites of polymer and organic or inorganic fillers, such as glass, carbon and cellulose fibers for improved mechanical strength, and ceramic fillers to increase abrasion resistance, such as corundum [21].

Nevertheless, the range of commercially available FFF filaments with approval for medical applications is still limited. High-performance plastics based on polyetherketones, namely PEEK and PEKK, are particularly common for medical applications. For these materials, implant-grade filaments have been available for several years [22,23]. For skin contact and short-term contact with body fluids, the high-performance polymer polyphenylene sulfone (PPSU) is available, also with barium sulfate filling for better visibility during X-ray or CT examinations [22]. A polyamide polyolefin cellulose filament (PAPC) is approved for short-term use in the body and has an inherent visibility on X-rays [24]. For Class 1 medical devices, i.e., products with temporary use and a low degree of invasiveness, various certified filaments are available on the market, such as ABS, PETG, elastic thermoplastic polyolefin (TPE), and PLA, which is also sold as an antibacterial version with copper nanoparticles. In addition, a whole range of approved resorbable materials in filament form are available for FFF. These include PLA, polycaprolactone (PCL), polyglycolic acid (PGA), and polydioxane, as well as copolymers of these materials with tailor-made degradability in the body and thus the possibility to personalize the object and material simultaneously.

In contrast, polyolefins, including polyethylene (PE), polypropylene (PP), and polybutene-1 (PB-1), are still seldom used in extrusion-based 3D printing, even though they account for about half of the global plastics production [25]. Polyolefins, which are high-molar mass hydrocarbon materials, have a wide range of applications worldwide and represent a cost-effective and resource-efficient material, also for medical applications [26]. Syringes, body fluid containers, tubes, instruments, and closures are examples of medical polyolefin products that are valued for their degree of purity and low allergy potential. A high-molecular-weight PE is also used in high-performance applications such as acetabular implants, indicating the long-term biocompatibility [27]. The success of the materials is based on a balanced range of properties in terms of tailored mechanics and processability, low density, low water absorption, and chemical resistance to common polar solvents and many acids and bases [28,29,30]. In addition, the often solvent-free catalytic synthesis of polyolefins is highly cost-, resource-, and energy-efficient [25]. The feedstock can be obtained from fossil as well as renewable sources. In combination with an easy recyclability as materials and as sources of chemical feedstocks and energy, polyolefins become the leading polymers in Landis’ life cycle assessment, ahead of biopolymers [31]. Furthermore, polyolefins will have a potential in the upcoming molecular recycling due to their hydrocarbon nature. Industrial approaches such as MoReTec technology from LyondellBasell enable the safe and sustainable recycling of contaminated plastic waste into secondary raw materials, from which in turn new, high-purity polyolefins suitable for medical applications can be produced highly efficiently [32].

Despite its economic success and its sustainability potential, 3D printing of semi-crystalline polyolefins such as PE or PP is currently still associated with numerous difficulties. Warpage related to the thermal shrinkage of these materials, which results from the crystallization during the cooling process, is one of the main challenges in case of additive processing [33]. A further challenge presents the use of appropriate printing platforms, as polyolefins only adhere to few surfaces made of polyolefins or polyolefinic rubbers due to their low surface energy [6,7].

## 4. Optimization of Polyolefins for Extrusion-Based AM

To overcome the intrinsic warpage challenge, many attempts were made to reduce crystallinity and shrinkage by ethylene-propylene copolymers [34]. The vast majority of available olefinic filaments for FFF is sold under the name polypropylene, but belongs to the group of copolymers. However, the positive effect of reduced warpage of ethylene-propylene copolymers is accompanied by a reduced stiffness and a significantly reduced heat deflection and melting temperature, thus limiting the range of applications. In particular, steam sterilization at 121 °C is generally not possible, which makes cost- and time-efficient use in the medical field much more difficult.

The use of fillers of different aspect ratios and morphology, such as glass fibers or beads, carbon fibers, silicate, and calcium carbonate, is another common approach to reduce warpage in polyolefins while improving the physical and mechanical properties [6,35]. In all cases, uniform dispersion of the filler in the PP matrix is essential to reduce thermal shrinkage. Thereby, the particle size of the fillers has an impact on the warpage tendency of printed PP. Spherical perlite fillers with a dimension of 20 µm showed a positive effect on the reduction of shrinkage and warpage of PP in FFF, which could not be achieved to the same extent with larger particles with an expansion of 100 µm [36].

Another approach to reduce warpage in polyolefins is the optimization of printing and process conditions. Carneiro et al. [6] established the fundamental basis for the dimensional accuracy of neat PP through FFF process control in terms of optimized printing parameters, printing path, and improved build plate adhesion. However, the printed specimens exhibited up to 30% loss of mechanical performance as compared to compression molded specimens of the same material. Based on this work, various groups showed how different process strategies, such as optimized extrusion and chamber temperatures, deposition strategies, optimized build plate materials, or the use of pre-deformed computer-aided design (CAD) files, can improve both warpage and mechanics of PE and PP [7,33,37,38,39].

**Figure 2:**
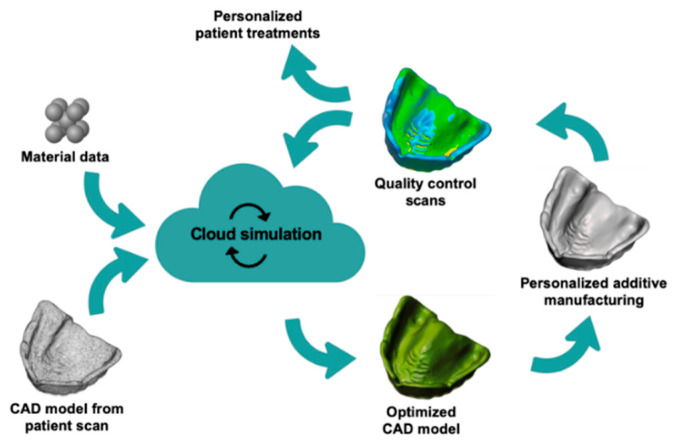
Implemented workflow for producing personalized medical tools through additive manufacturing and integrated self-learning simulation to overcome material-based warpage challenges. CAD: computer-aided design.

In connection with medical applications, even higher demands are required on process-controlled warpage optimization. For example, it must be ensured that no internal stress remains in the material, which would be released at elevated temperatures during steam sterilization causing deformations. In order to achieve this reliably and efficiently for a wide range of medical applications, not just for polyolefins, a digital workflow is essential and would open up new opportunities. In the medical sector, digital workflows are used, for instance, to plan guided surgeries based on CBCT scans to obtain predicable results. In addition, the computer-aided design/computer-aided manufacturing (CAD-CAM) process helps in the reproducible production of objects, which are subjected to quality control. This digital workflow, which focuses on AM, commences with data acquisition using 3D scanners or conversion of 3D radiographical images into objects (Figure 2). Second, patient-specific medical applications are designed (CAD). Third is the material- and object-specific preparation of the digital 3D data, concerning distortion and print path optimization. Fourth are additive or alternatively subtractive manufacturing methods. Finally, the digital workflow is complemented by quality control, providing data for continuous improvement of the process.

This digital process is still comparatively complex because it usually requires a lot of personnel and thus limits its use in everyday clinical practice. In the future, a self-learning automatic simulation process will play a key role, as it optimizes the 3D printing process with personalized CAD objects based on predefined application scenarios. Through cloud computing, the calculation power required for this is no longer a limitation, and the global input enables continuous improvement of processes. However, despite the potential of digital optimization for AM, the printing material is the pivotal element. The material defines the framework of possibilities in terms of geometry and physical and chemical performance and is the keystone for the reliability of the final 3D printed object.

Polyolefins, and PP in particular, have promising potential as an emerging standard material in AM of medical and assistive devices. With a wide variety of medical devices and aids already being made from this material, it has gained a high level of acceptance among medical staff, patients, and regulatory authorities alike. This acceptance and reliability are the basis for the increasing use of PP in medical AM. The goal should be to create a high degree of compatibility between conventionally manufactured and 3D-printed parts. Medically certified and FFF-optimized PP grades are characterized by a wide process window and a low melting point compared to typical medical printing materials such as PEEK or PEKK. As a result, this class of hydrocarbon materials can be processed on any standard FFF printer and the printing results are reliable and comparable with products manufactured by injection molding, even with fluctuations in process control, which is particularly common with rather simply constructed but mass-market FFF printers. Thus, 3D printing of PP has the potential to complement conventional manufacturing techniques to respond flexibly to peaks in demand, supply bottlenecks, or personalized application profiles without requiring adaptation of existing processes by doctors or patients.

## 5. Extrusion and Application of a Medical-Grade Polyolefin Filament

**Figure 3:**
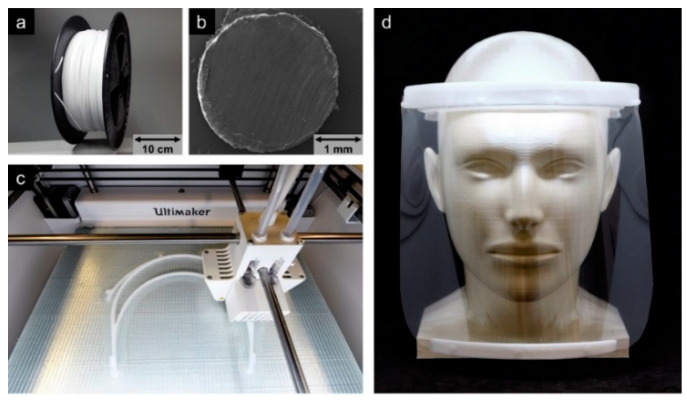
Extruded polypropylene (PP) filament on a spool (**a**) and SEM image of the cross-sectional area of the filament (**b**) applied for 3D printing of sterilizable and reusable headbands for face shields (**c**,**d**) during the COVID-19 pandemic for use as protective equipment at the medical center Freiburg, Germany, manufactured from a PP with certified biocompatibility (USP class VI).

**Figure 4:**
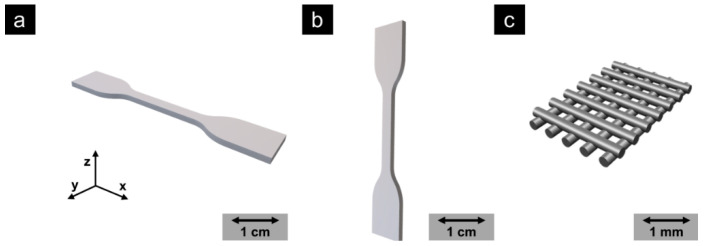
Illustration of the different printing orientations for the mechanical characterization: (**a**) x-y printing; (**b**) x-z printing direction; (**c**) illustration of the crossed linear ±45° 3D printing infill orientation used for reliable mechanical properties.

Considering this background, a PP with certified biocompatibility (USP class VI, Chapter 88 [40]) was successfully used by our group as material for FFF printing to produce PPE at the highest medical standards for our university hospital. For this purpose, an optimized digital workflow based on warpage simulations has already been successfully applied. During the beginning of the COVID-19 pandemic in Europe in spring 2020, a large number of face shields for medical staff were needed. The original CAD templates for the face shields were provided by the printer manufacturer, Prusa [41]. For the 3D printing of the headbands (Figure 3c), a novel PP copolymer (Healthcare PP, Purell type, LyondellBasell Industries B.V. (Rotterdam, The Netherlands), ρ = 0.9 g mL^−1^) was used, allowing a wide temperature range for the application. The PP exhibits excellent layer bonding properties in all three dimensions (Table 1 and Figure 4). Due to the purity and certified biocompatibility of the PP, irritation in case of direct contact of the PP with skin or wounds is widely excluded, even for extended wearing periods. The flexibility of the PP (Table 1) allows to adapt the headbands to the head size to further improve wearing comfort. Additionally, the PP headbands are resistant to common disinfectants and are steam sterilizable due to its high Vicat softening temperature, enabling sustainable and hygienic recycling in the clinical environment.

**Figure 5:**
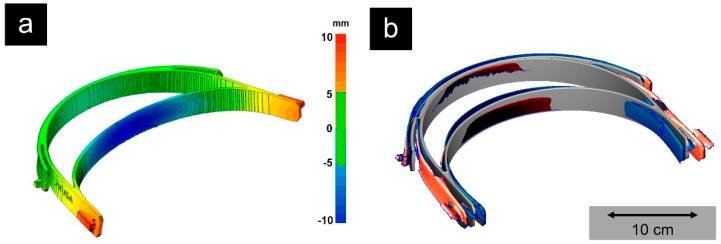
The predicted deformation by simulation after the 3D printing process based on the original geometry (**a**). 3D scan of the printed medical-grade polypropylene headbands based on the original Prusa templates (red) and based on the optimized template (blue) in comparison to the original STL file (grey) (**b**). The optimized headbands exhibited a better fit by compensating for the warpage and could be printed in large numbers without faulty rejects due to the process optimization.

**Figure 6:**
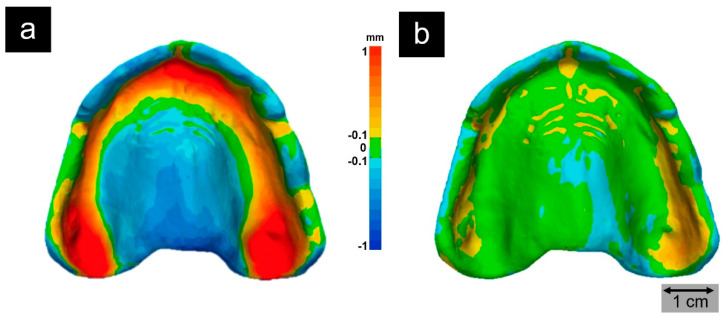
Target-actual comparison of the intaglio surface of a FFF printed denture base made of medical-grade PP applying standard PP printing conditions (**a**) and a warpage-optimized PP with reduced crystallinity after 3D printing process optimization in terms of pre-deformed, warpage-compensating CAD files and temperature management (**b**). Printed denture bases were digitized (VL 500, Keyence, Osaka, Japan) and compared to the original STL file with an inspection software program (Geomagic Control X, 3D Systems, Rock Hill, SC, USA). The data of the intaglio surfaces was superimposed with the data of the original by using a local best-fit algorithm according to Gauss. Subsequently, a 3D surface comparison was carried out and the discrepancies were illustrated using a color map with a tolerance of ±100 µm (green).

The PP filament was produced on a twin-screw extruder (COLLIN TEACH-LINE™ ZK 25 T, 180 °C, 45 rpm, Figure 3a) with a die having a diameter of 3.3 mm, a water-cooling system, and a winding unit (take-off speed 90 mm s^−1^). The resulting filament exhibited dimensions of 2.8 × 2.6 (± 0.05) mm and was used in a fused filament fabrication (FFF) printer (Ultimaker S5, Ultimaker B.V., Utrecht, Netherlands; Figure 3b). The printer was equipped with a steel nozzle (0.8 mm diameter) and fiber-reinforced PP adhesive tape (Scotch extreme packaging tape, 3M, Saint Paul, MN, USA) as the print bed. Printing was performed at a nozzle temperature of 210 °C, build plate temperature of 60 °C, printing speed of 50 mm s^−1^, layer height of 0.2 mm, and 100% infill. The printer settings resulted in a print time of 2.5 h for each headband exhibiting a mass of 40 g, whereby three headbands could be printed simultaneously on one 3D printer. This allowed several hundred face shields to be produced decentrally for the hospital and other local facilities in a short time. To compensate for the inherent warpage issues of the material, thermomechanical calculations were carried out simulating the printing process based on the physical properties of the medical-grade PP and its melt using the software Digimat-AM and the Finite Element solver Marc from MSC software. The input for simulating the process comprised the part geometry; thermomechanical material properties; the 3D printing parameters, such as the nozzle, build plate, and chamber temperature; the printing pathway; and the cooling conditions. The computational mesh was made of cubic voxels exhibiting an edge length of 0.6 mm, leading to about 220,000 elements for the headband. The resulting deformation of the headband as obtained from the simulation process was subtracted from the original target geometry to generate a counter-warped CAD file. The resulting 3D-printed PP headbands exhibited an optimal fit on the one hand and a printing process that was so reliable that the reject rate was reduced to zero (Figure 5). Organic forms such as denture bases pose a greater challenge in AM. In this context, our first tests using material and process optimization showed a considerable reduction in warpage and shrinkage when printing polyolefins (Figure 6).

## 6. Validation of the Process Chain

In order to apply the novel PP filament in FFF as a standard for the production of medical devices, the entire process chain must be evaluated and validated. This includes all steps from feedstock production and design phase to the final stage, when it needs to be ensured that the additively manufactured product does not harm the user or patient. The international standard ISO 9001:2015 [43] generally defines the requirements for quality management along the supply chain, whereas ISO 13485:2016 [44] specifies the requirements for regulatory purposes of medical devices. Organizations offering AM as a service in the medical field should incorporate these and other relevant quality standards to provide the basis for proper process documentation and quality management. Certification of the process, however, is performed by national certification bodies and auditing companies. If the auditing was successful, a recommendation for approval is issued for this organization. In the USA, governing agencies such as the Federal Aviation Administration (FAA) and the Food and Drug Administration (FDA) deal with the qualification of medical devices. In Europe, products must be certified by an accredited body to conform to relevant standards, i.e., the Conformité Européenne (CE), before they can be sold in the market [45].

Even though our products are manufactured in-house and are primarily intended for use at the university hospital during pandemic times, the process chain must be validated in order to achieve successful certification. First, the raw materials for 3D printing should be evaluated as for any other manufacturing process, with adequate quality control to guarantee a homogeneous and traceable manufacturing substrate [45]. Furthermore, technical devices and settings need to be considered and documented for regulatory purposes. This includes the specification of the extruder used for the production of the filament as well as the desktop 3D printer for the printing process. Moreover, printing parameters such as layer height, nozzle temperature, and the temperature of the printing bed need to be described and constantly monitored during the printing process, since they have a considerable impact on the physical characteristics of the final device [46]. In addition, all components that come into contact with the medical device or the material used to print it, such as the filament feeder, the nozzle, and the build plate, are also subject to strict regulations. Quality measurements of the printed objects are necessary to ensure consistency between the printed devices [47]. After printing, post-processing of the manufactured objects is necessary and needs to be defined; thus, FFF-printed objects require, for example, the removal of possible support structures. To obtain the final crystallization and reduced warpage of the final product, our printed objects made of PP were tempered at 80 °C for 24 h, leading to an increasing degree of crystallinity from 33% to 35%, as calculated from the melting enthalpy obtained from differential scanning calorimetry. It needs to be validated that the postprocessing does not alter the mechanical properties in a negative way. Furthermore, biocompatibility needs to be certified, especially if the final products are used in the medical field. Although the PP copolymer used for the extrusion of the filament has a certified biocompatibility (USP Class VI), material properties can be altered by the 3D printing process. Therefore, a biological evaluation of medical devices according to the international standard ISO 10,993 [48] is necessary. Especially in terms of biocompatibility, new questions arise in the field of 3D printed objects [49]. First observations of our ongoing biocompatibility tests evaluating the printed PP specimens (Healthcare PP, Purell type, LyondellBasell Industries B.V.) on human cell lines were very promising. The hospital-based, in-house manufactured items need to meet the same standards of safety and efficacy for medical products as manufactured elsewhere. Thus, it is necessary to validate how the printed objects can be sterilized while maintaining their dimensional accuracy without negatively changing their mechanical properties [50]. In general, sterilization of medical products can be achieved by autoclaving, ionizing radiation, dry heat, or heat/chemical vapor [51]. Thereby, the moist heat in autoclaving or steam sterilization is a widespread, cheap, and effective form of sterilization [52]. However, it was observed that steam sterilization of specimens manufactured with a liquid photopolymer may lead to a deformation of the printed objects [53]. Other investigations of biocompatible resin materials, however, revealed no major deformations or structural changes after steam sterilization at a temperature of 121 °C [50,52]. Surgical guides printed in the FFF manufacturing process with a printed biopolymer also exhibited a high accuracy after steam sterilization [16]. In contrast to most commercially available but not medical-grade ethylene–propylene copolymers used for FFF, the medical-grade PP used for PPE and other medical devices by our group is steam-sterilizable due to its high Vicat softening temperature. Ongoing investigations by our group are evaluating the influence of printing parameters as well as different material components on the dimensional stability of FFF-printed polyolefins before and after steam sterilization. Further studies are stipulated to assess whether the printed objects have been sufficiently disinfected after sterilization or whether different printing requirements, e.g., sterile conditions, are required. The inside of the printed objects must also be examined if the printed instruments or aids are used in the surgical field or if the printed parts are manually modified (e.g., by grinding).

## 7. Future Perspectives

Once a robust, comprehensive, and validated process chain for unfilled medical-grade PP has been established, the process could serve as a digitized blueprint for a wide range of applications for various 3D printing materials and material systems in the medical field. Due to the ongoing pandemic, the demand for medical products and PPE that can be produced at short notice and on site will continue to increase. In addition, personalized patient care based on 3D printing will become increasingly important for better, more comfortable, and more economical treatment of patients with specific needs that are not adequately addressed by conventional therapy techniques.

In our group, the main focus to meet these emerging demands is set on sustainable and recyclable polyolefins, which can be enhanced with fillers and additives to access a wide range of applications with desktop 3D printers. Selected medically approved fillers provide opportunities to highly abrasion-resistant and highly stiff materials which, in combination with FFF, represent an outstanding potential for temporary, cost-effective dental restorations. Furthermore, polyolefins with functional additives for FFF enable the production of personalized self-cleaning and bacteria-repellent equipment, for example, for dental medicine and surgical applications. This may considerably reduce the number of medical complications. Also promising in medical filament-based 3D printing is the use of functional multi-material systems that offer customized functionality driven by external triggers, thereby enabling personalized treatment processes on and in the patient. The harmonized combination of digital workflow, filament-based 3D printing, and customized materials therefore holds great promise in improving patient care and global access to medical protection and care, and will consequently play an important role in the fight against and the prevention of future pandemics.

## Figures and Tables

**Table 1:** Properties of the medically certified polypropylene used to print protective equipment.

Parameters	Value
MFR (230 °C/2.16 kg) ^a^	14.9 ± 0.7 g (10 min)^−1^
T_m_^b^	165 °C
VST A/50 ^c^	142 °C
Degree of crystallinity ^d^	33% ^e^/35% ^f^
Tensile modulus ^g^	(1.00 ± 0.12) ^h^/(0.83 ± 0.08) ^i^ GPa
Tensile strength ^g^	(19.85 ± 0.04) ^h^/(18.1 ± 0.2) ^i^ MPa
Elongation at break ^g^	(15 ± 2) ^h^/(8.0 ± 1.4) ^i^ %

^a^ MFR (melt-mass flow rate), according to ISO 1133-1; ^b^ DSC (differential scanning calorimetry) second heating cycle (10 K min^−1^); ^c^ VST (Vicat softening temperature), according to ISO 306; ^d^ calculated from the melting enthalpy obtained from DSC second heating cycle (10 K min^−1^) based on 100% crystalline polypropylene [42]; ^e^ obtained from 3D printed specimens immediately after the printing process; ^f^ obtained from 3D-printed specimens after annealing at 80 °C for 24 h; ^g^ according to DIN EN ISO 527-2/5A using six simultaneously 3D-printed specimens each; ^h^ printed in x-y direction using 100% infill and crossed linear ±45° orientation; ^i^ printed in xz direction using 100% infill and crossed linear ±45° orientation.
